# 

**Published:** 1860-03

**Authors:** 


### LIST OF COLLABORATORS.

ARMOR, S. G., M.D.,
Late Professor of Pathology and Clinical Medicine, Missouri Medical College.

ATLEE, JOHN L., M.D.,
Late President Pennsylvania State Medical Society.

ATLEE, WALTER F., M.D.,
Philadelphia.

BARCLAY, ROBERT G., M.D.,
Jerusalem, Syria.

BELL, JOHN, M D.,
Philadelphia, formerly Professor of Medicine, Medical College of Ohio.

BEMISS, S. M., M.D.,
Professor of Clinical Medicine, University of Louisville.

BLACKBURN, L. P., M.D.,
Natchez, Miss.

BLACKIE, GEORGE C., M.D.,
Professor of Botany, University of Nashville.

BLACKMAN, G. C., M.D.,
Professor of Surgery, Medical College of Ohio.

BOBBS, J. L., M.D.,
Formerly Professor of Anatomy, Indianapolis.

BOWEN, W. S., M.D.,
New York.

BOZEMAN, N., M.D.,
Montgomery, Ala.

BRADFORD, J. T., M.D.,
Augusta, Ky.

BRINTON, JOHN H., M.D.,
Lecturer on Operative Surgery, Philadelphia.

CARNOCHAN, J. M., M.D.,
Professor of Surgery in the New York Medical College.

CHIPLEY, W. S., M.D.,
Professor of Medicine, Transylvania University.

CLAPP, A., M.D.,
New Albany, Indiana.

CLIFFE, D. B., M.D.,
Franklin, Tenn.

DA COSTA, J., M.D.,
Lecturer on the Practice of Medicine at the Philadelphia Medical Association.

DICKSON, SAMUEL H., M.D.,
Professor of Medicine, Jefferson Medical College.

DUNGLISON, RICHARD J., M.D.,
Philadelphia.

DUPIERRIS, M., M.D.,
Havana, Cuba.

EDGAR, W. F., M.D.,
United States Army.

FARRAR, S. C., M.D.,
Jackson, Miss.

FENNER, C. S., M.D.,
Memphis, Tenn.

FISCHER, EMIL, M.D.,
Philadelphia.

FLINT, AUSTIN, M.D.,
Professor of Clinical Medicine, Auscultation, and Percussion, New Orleans School of Medicine.

FRICK, CHARLES, M.D.,
Professor of Materia Medica, University of Maryland.

GADBURY, W. Y., M.D.,
Benton, Miss.

GIBBS, O. C., M.D.,

Chautauque County, New York.

HALL, J. P., M.D.,
Glasgow, Ky.

HAMMOND, WM. A., M.D.,
United States Army.

HARDIN, JOHN, M.D.,
Professor of Obstetrics, Kentucky School of Medicine, Louisville.

HARTSHORNE, EDWARD, M.D.,
Surgeon to the Pennsylvania Hospital.

HASKINS, E. B., M.D.,
Professor of Medicine, Shelby Medical College, Nashville, Tenn.

HENTZ, C. A., M.D.,
Marianna, Florida.

HEWSON, ADDINELL, M.D.,
One of the Surgeons to Wills Hospital for Diseases of the Eye, Philadelphia.

HOLLINGSWORTH, SAMUEL L., M.D.,
Formerly Editor of the Medical Examiner, Philadelphia.

JEWELL, WILSON, M.D.,
Philadelphia, formerly President of the Quarantine and Sanitary Convention.

KEATING, WM. V., M.D.,
Lecturer on Obstetrics and the Diseases of Women, at the Philadelphia Medical Association.

KERR, JOHN G., M.D.,
Canton, China.

KUNKLER, G. A., M.D.,
Madison, Ia.

LOGAN, BEN., M.D.,
Shreveport, La.

LOPEZ, A., M.D.,
Mobile, Ala., formerly President of the Alabama State Medical Society.

MEIGS, J. AITKEN, M.D.,
Professor of the Institutes of Medicine in the Medical Department of Pennsylvania College.

MITCHELL, S. WEIR, M.D.,
Lecturer on Physiology at the Philadelphia Medical Association.

MOREHOUSE, GEORGE, R., M.D.,
Philadelphia.

RAPHAEL, B. I., M.D.,
New York.

REEVE, J. C., M.D.,
Dayton, Ohio,

RIVES, L. C., M.D.,
Formerly Professor of Midwifery, Medical College of Ohio.

SMITH, J. LAWRENCE, M.D.,
Professor of Chemistry, University of Louisville.

SNEED, W. C., M.D.,
Frankfort, Ky., formerly President of Kentucky State Medical Society.

SPILMAN, C. H., M.D.,
Harrodsburg, Ky., formerly President of Kentucky State Medical Society.

STORER, HORATIO R., M.D.,
Formerly one of the Physicians to the Boston Lying-in Hospital, Boston, Mass.

SUTTON, GEO., M.D.,
Aurora, Ia.

SUTTON, W. L., M.D.,
Georgetown, Ky., formerly President Kentucky State Medical Society.

THOMPSON, S., M.D.,
Albion, Ill., formerly President of the Illinois State Medical Society.

TODD, L. BEECHER, M.D.,
Lexington, Kentucky.

WILLIAMS, E., M.D.,
Cincinnati, Ohio.

### Bi-Monthly Bibliographical Record.

AMERICAN.

Footfalls on the Boundary of Another World; with Narrative Illustrations. By Robert Dale Owen. 12mo., pp. 528. J. B. Lippincott & Co.: Philadelphia, 1860. (From the publishers.)

A Practical Treatise on Fractures and Dislocations. By Frank Hastings Hamilton, M.D., etc. 8vo., pp. 757. Blanchard & Lea: Philadelphia, 1860. (From the publishers.)

A Medico-Legal Treatise on Malpractice and Medical Evidence; comprising the Elements of Medical Jurisprudence. By John J. Elwell, M.D., etc. 8vo., pp. 588. New York: John S. Voorhies, 1860. (From the author.)

Introductory Lectures and Addresses, on Medical Subjects, delivered chiefly before the Medical Classes of the University of Pennsylvania. By George B. Wood, M.D., etc. 8vo., pp. 460. J. B. Lippincott & Co.: Philadelphia, 1859. (From the publishers.)

The Diagnosis, Pathology, and Treatment of the Diseases of the Chest. By W. W. Gerhard, M.D., Fourth edition. 8vo., pp. 448. Philadelphia: J. B. Lippincott & Co., 1860. (From the publishers.)

Observations on Some of the Physical, Chemical, Physiological, and Pathological Phenomena of Malarial Fever. By Joseph Jones, M.D., etc. Reprint, 8vo., pp. 419. Philadelphia, 1859. (From the author.)

An Epitome of Braithwaite's Retrospect of Practical Medicine and Surgery. By Walter S. Wells, M.D. Part i. 8vo., pp. 304. New York, 1860. (From the author.)

Suggestions on Medical Education; an Introductory Lecture delivered in the Medical College of Georgia. Pamphlet, pp. 26. Augusta, 1860. (From the author.)

An Essay on Hernia. By James Bryan, M.D., etc. Part i. 4to., pp. 14. Philadelphia: F. J. Pilliner, 1860. (From the author.)

Contributions to Operative Surgery and Surgical Pathology. By J. M Carnochan, M.D., etc. Part iii. 4to., pp. 81–127. Philadelphia: Lindsay & Blakiston, 1860. (From the publishers.)

Valedictory Address to the Anatomical Class of the Philadelphia School of Anatomy. By D. Hayes Agnew, M.D., etc. Pamphlet, pp. 20. Philadelphia, 1860. (From the author.)

Inaugural Address, delivered in the Medical Department of the Pennsylvania College. Pamphlet, pp. 23. Philadelphia, 1859. (From the author.)

Selections from a Report on Ovariotomy; read before the Kentucky State Medical Society. By J. Taylor Bradford, M.D. Pamphlet, 8vo., pp. 56. (From the author.)

The Subjective and Objective Influences of Medicine; an Introductory Address. By E. B. Haskins, M.D. Pamphlet, pp. 23. Nashville, 1859. (From the author.)

FOREIGN.

Clinical Lectures on the Principles and Practice of Medicine. By James Hughes Bennett, M.D., F.R.S.E., etc. 8vo., pp. 952. W. S. S. & W. Wood: New York, 1860. (From the publishers.)

On the Diseases and Injuries of the Joints: Clinical and Pathological Observations. By Thomas Bryant, F.R.C.S., etc. Post 8vo., pp. 273. London: Churchill, 1859. (From the publishers.)

On Excision of the Knee Joint. By Oliver Pemberton. Reprint, 12mo., pp. 32. London, 1859. (From the author.)

Clinical Lectures on certain Acute Diseases. By R. Bentley Todd, M.D., etc. 8vo., pp. 308. Philadelphia: Blanchard & Lea, 1860. (From the publishers.)

Notes on Nursing: What it is, and what it is not. By Florence Nightingale. 8vo., pp. 79. London, 1860.

On the Injurious Effects of Mercury in the Treatment of Disease. By S. O. Habershon, M.D., etc. Post 8vo., pp. 86. London: Churchill, 1859.

On Diseases of the Throat, Epiglottis and Windpipe, etc. By Geo. D. Gibb, M.D., etc. Fcap. 8vo. London: Churchill, 1859.

A Treatise on Obscure Diseases of the Brain and Mind; their Incipient Symptoms, Pathology, Diagnosis, Treatment, and Prophylaxis. By Forbes Winslow, M.D., etc. 8vo., pp. 60. London: Churchill, 1860.

Lehrbuch der Augenheilkunde. By Joseph Pilz. 8vo., pp. 1024. Prague: André, 1859.

Des Déviations des Dents et de l'Orthopédie Dentaire; Des Soins a Donner aux Enfans a l'Époque de la Seconde Dentition. By F. Lefoulon. 8vo., pp. 56. Victor Masson: Paris, 1859.

Religious Revivals in relation to Nervous and Mental Diseases. By T. Stevenson Bushman, M.D., etc. 8vo. London: Churchill, 1859.

